# A Confession and a Warning

**Published:** 1890-01

**Authors:** E. W. Berridge

**Affiliations:** London


### A CONFESSION AND A WARNING.

E. W. Berridge, M. D., London.

We are none of us infallible, not even the youngest of us; and much may be learnt from our failures and errors, if we will only have the manly candor to admit and point out our own shortcomings, that our colleagues may learn to avoid them also. There are, of course, some who are never wrong, at least in their own estimation. To this class belong the physicians who, while pretending to be Hahnemannians, declare that in certain cases of severe pain anæsthetics must be resorted to; the cause being, in their opinion, not their own fallibility in the selection of the *simillimum*, but a supposed imperfection in Homœopathy itself. An instance of this conceited egotism occurred a few years ago, where a physician, after finding that a single dose of a high potency failed to relieve severe pain, in fifteen minutes, immediately lost heart, if not his head, and proceeded to give an anæsthetic; and though high potencies subsequently relieved this very patient of pain quite as severe, instead of admitting the bare possibility that he had not selected the *simillimum*, declared "I am now convinced" [Query, from the evidence of one case ?] "that there are cases, though I have found them few and far between, where the palliative measures must be adopted." Will it be believed that *only two or three years after* this occurrence, this same physician had the unblushing effrontery to declare, "I state it as fact, that *during the past twelve years* I have *never once* prescribed * * * an opiate or allopathic palliative." What confidence can be placed in the assertions of a physician who so flatly contradicts himself in his endeavor to exalt himself above Hahnemann, and yet to find favor in the sight of Hahnemann's disciples?

Mrs. —— was safely delivered of her first child Sept. 30th, 1883. About the beginning of October she began to suffer much from piles, for which the physician who delivered her prescribed *Sepia*$^{200}$, which did no good. I was then consulted by letter on October 16th and prescribed *Phosphorus*$^{cm}$ (F.C.), which much relieved her, entirely removing the following symptom, "When sitting or reclining, shooting pains like fine darting needles in vagina near orifice; they come on suddenly like several beats in succession, then stop and begin again after a measured interval; they come without apparent cause four or five times daily, continuing with their short intervals of cessation for ten or fifteen minutes; a slight change of position seemed to give relief at times; needle-pains in rectum also near orifice, sometimes alternating with the vaginal pains, but more often independent."

On October 28th I was telegraphed for. She had been riding in her carriage, and the cold wind had blown on her right side, causing sciatica. I found her with constant dull pain on outside of right hip as if it had been hammered; worse by lying on left side, better by lying on right side and by hot applications. Intermittent shooting pain from right hip to knee and toward centre of abdomen. Cramp-pains in right calf and back of right thigh when stepping on right foot or stretching out the right leg. Her attendant physician had given her *Arsenic* and *Colocynth*, which were "like so much water on a duck's back." I prescribed *Ammonium-carb.*$^{2300}$ (Jenichen) every two hours till better.

On November 4th reported pains in the hip better after first dose; after further improvement she continued the medicine at longer intervals. Now the hip pains are gone.

December 7th.—Her attendant physician sent me the following report: Since November 22d she had suffered intensely from piles, and was getting worse, in spite of the *Carbo-animalis* and *Natrum-carb.* Examination showed three piles *very much inflamed*, the smallest the size of a pea; the largest almost the size of the end of the thumb, and situated nearest the vagina. On pressing upward on this largest pile on the side next the anus, there is felt a knife-pain in pile and extending far up rectum. This pile has a hard internal kernel, and shrivels on touch. Feels weak, nervous, despondent, and utterly incapable of doing anything. After stool, excessively sharp pain like a knife cutting; she walks for relief, and it is better from hot fomentations. Numbness of right leg, with feeling of a tight ligature just above knee, and feeling of a large ball pressing in just below hip at back of thigh; this makes her walk lame, and right leg feels shorter than left. These leg symptoms were at first felt only before and after stool, but now continue even when there is no stool. Stool of waxy consistency, gray, comes down and then stops, causing sharp, cutting pain. Hands and feet become suddenly cold. Before and after stool, coccyx feels pushed out with burning. Pain in piles after stool, *relieved by lying on right side.* Enema of water at temperature of 100° is of but little use; the anus seems constricted, so that it is painful and difficult to pass the enema. Had a slight stool yesterday evening from the enema, and the subsequent pain was agony, lasting till this afternoon. Rectum seems inactive, as if the stool escaped only by gravitation; the stool passes slowly, and does not all pass, but some remains behind in rectum. Sensation of utter mental incoherence, thoughts are not ready, and expression labored; the incoherence is with regard to grammar; she says it is as if she were feeling her way in a foreign language. She says she suffers more pain than in her confinement, *and she wants chloroform.*

Now surely here was a case where I should be told that "something must be done," and "where the suffering was so great that it would be inhuman to have withheld a means of relief" which those physicians who live, move, and have their being in the late Sir James Simpson, "know from long (allopathic) experience if the agent was not incompatible with recovery." But being a Hahnemannian, though not claiming to be infallible, I preferred to select the homœopathic remedy in order to cure, rather than give "an agent which was not incompatible with recovery." I sent *Kali-carbonicum*$^{cm}$ (F. C.), a dose to be taken at once, and repeated every twelve hours. The first dose was taken on December 8th.

December 10th.—Patient came up to London, and called on me. No pain to-day on pressing pile. Less lameness on walking this morning. No stool to-day. Mental state continues.

*Kali-carb.*$^{3\,cm}$ (Fincke) every twelve hours.

December 11th.—Last evening, after the first dose of the new potency, used an enema of warm olive oil; constriction of anus very much less, and no pain or difficulty with enema. Stool after enema was painful, but nothing like the former pain. The pains before and after stool of the same character, but less intense. No lameness or other leg symptoms after last stool. For thirty minutes, no pain after stool, but on pressing the pile the cutting returned, lasting some time. Stool still slow; rectum inactive, does not seem to expel all its contents, as if the stool only passed by force of gravitation., No coccygeal pains in connection with last stool. Hands and feet less cold. Incoherence less yesterday, and still less to-day. Less despondency.

*Kali-carb.*$^{em}$ (F. C.) every twelve hours.

December 13th.—Patient tells me that the burning pain referred to on November 28th, which had continued getting worse, improved after the first two doses of *Kali*, and is better now. Constriction of anus not returned; can introduce enema easily. The knife-pain felt as the stool descended, which was getting quite unbearable till she commenced the *Kali*, is comparatively slight now. After stool, has the same kind of pain, but less. Piles less inflamed, and less pain on touching them. Mental symptoms better. This morning after stool had the ligature sensations, and the lameness, but no other leg symptoms, and even these were less than formerly. Feet cold at times. Action of bowels still incomplete, rectum seems to lose power at end of stool, but not so much as before coccygeal pains returned, but less. Feels decidedly better on the whole than two days ago.

*Kali-carb.*$^{cm}$ (F. C.) every twelve hours.

December 19th.—Says she "feels herself improving from hour to hour." Had a stool morning of 15th; concomitant symptoms the same in character, but infinitely less in degree; the itching, however, seems to increase; the leg symptoms were present, though modified; had to lie down only an hour or two. Every morning following had about two sharp knife-pains; no stool nor desire for it, and no other pains. Evening of 18th, another stool, some pain, but stool quite healthy. No mental symptoms. *Kali-carb.*$^{c}$ (F. C.) once daily for seven days.

December 27th.—Now I saw the fatal error I had committed in repeating the dose after so great an improvement. I should have allowed it to act. Patient's letter of to-day says that on

December 20th she had stool, and never were mental symptoms worse. On 21st another stool; the pain equaled anything she had ever suffered, and continued for thirty-six hours. Next stool on 24th not so painful. On 26th suffered agony, and at the time of writing, fourteen hours after the stool, the pain is at times excruciating. The sensation is no longer like a cutting knife, but more like pulse-beats and burning. There is a dead heavy ache all the time, and sensation as if the bowel protruded, the pulse-beats and burning being present at frequent intervals. The large pile does not appear to have the kernel it did, and there is no knife-pain on pressing it. No return of the leg symptoms. She says, "my faith and hope are entirely gone;" that she will give Homœopathy one more trial, and if there is no decided relief from pain, must seek it elsewhere. (Query, in *Chloroform?*)

Such was the result of too frequent repetition of the dose. I wrote to her to lie on her back with the hips raised, after stool; to retain the stool as long as possible, until there was a strong desire, and then use enema of warm olive oil. *Hamamelis*$^{cm}$ (Fincke) every four hours for six days. On studying this case again after the further experience of six years, I am not at all sure whether this was the best treatment. Perhaps it would have been better to have given no medicine, and let the aggravation pass off. It will be noticed that the relief from the *Hamamelis* was not so marked as from the first few doses of *Kali.* However I did not give her *Chloroform,* which is something to be looked back to with satisfaction.

December 31st.—Writes that early in morning of 29th, after several days constipation, there was a stool, not so painful. Later, on 29th, the medicine arrived. In evening, after taking two doses, a second stool came suddenly; there were pains in abdomen, as if she had eaten green fruit, though her diet had been as usual. There was great pain in the piles, with burning and throbbing afterward, so that she nearly fainted; profuse perspiration at the time, followed by chilliness; the pains lasted about eighteen hours. The large flabby pile was not at all sensitive; but under it, and extending beyond it like a half-moon around anus was something that felt hard and was very painful. The posterior pile had doubled in size since 29th. Feels in her head as she did when a girl, after being half-starved in a school in Germany; nails have ridges in them. This evening (Dec. 31st), there was another stool, followed by pain for about ten hours. The hard, tender swelling under the anterior pile has gone; less burning and throbbing, and only in posterior pile.

Says she thinks the medicine has helped her. Begins the last powder to-morrow. After finishing it, is to take *Hamamelis*$^{45m}$ (Fincke) three times daily for six days.

January 19th, 1884.—Was telegraphed for. Found she had had a relapse from catching cold, and had come up to London on a visit to a friend, a professed homœopathic physician. This doctor, though knowing that she was my patient, took her to consult a mongrel. The result of this consultation was, that the patient was ordered to prevent the bowels from acting. When I saw her she had not allowed herself to have a stool for eleven days, except a slight one on ninth day, which she could not restrain, though all this time there was great urgency. The doctor had given her several medicines in rapid succession, with only temporary relief. Further particulars I could not learn, as the doctor, annoyed at his failure, and at the patient sending for me, purposely absented himself during my visit, merely leaving a list of the medicines used. I found the patient lying on the couch, with frequent and great urging to stool, which was frequently passing in spite of all her efforts to prevent it. Feeling of a large mass low down in rectum, which felt paralyzed. Constant aching and burning in rectum, relieved by rubbing. *Stool feels as if it slipped back*, with frequent urging, and a little passing. Feels a fear of suicide. Ordered her to allow the bowels to act at once, and prescribed *Silicea*$^{cm}$ (F. C.) every three hours till relieved.

January 20th.—Took first dose yesterday at five P. M. At six P.M., soft, enormous stool, first part large in size; one knife-pain as first portion of stool passed, but very little afterward. The stool did not seem to slip back after the first dose, but there was a steady slow stool without exertion. About seven P. M., another similar stool. At nine A. M. to-day, a third stool, with a little knife-pain at first portion of stool, and rectum felt weaker. After this stool, the external piles, which had vanished, re-appeared, and the rectum felt all loose and flopping about, as if it would prolapse on walking; this became better in course of day. In evening, another stool. Mental condition better. To-day, since early dinner, on lying down, hollow feeling in left chest, as if heart would stop, with feeling of impending danger, relieved by sitting up (never had this symptom before). Very sleepless for last two nights. Burning and throbbing in piles after stool, but decreasing each day. Urine only passes when straining at stool; no desire for it, and it comes without force; this is rather better to-day. Last two nights, when awake, creeping on back of hands. *No more medicine.*

March 6th.—Weaned baby this morning, and consulted me for trouble with the breasts, which *Bellad.*$^{cm}$ (F. C.) quickly relieved. Says that after taking the *Silicea* the bowels moved painlessly every day, and she has never had any real pains since, though the piles have not quite gone.

This case shows,

(1) The extreme danger of a too frequent repetition of the high potency.

(2) That even in the severest pains it is unnecessary to give *Chloroform* as an anæsthetic, our law being infallible.

(3) That patients who leave a Hahnemannian for a mongrel physician have to pay the penalty of increased suffering, in addition to the mortification of being compelled to return to their first love.

